# Proton-pump inhibitor use in combination with a CDK4/6 inhibitor: is formulation the issue?

**DOI:** 10.1093/oncolo/oyag293

**Published:** 2026-07-31

**Authors:** Calum Polwart

**Affiliations:** South Tees Hospitals NHS Foundation Trust, James Cook Cancer Institute, Middlesborough TS4 3BW, United Kingdom

I read with interest the article by Chen et al., describing their real-world observations of the outcomes of patients who received CDK4/6 inhibitors in combination with a proton pump inhibitor (PPI) in comparison to those who did not.^1^ Their findings raise the concern that outcomes may be reduced in patients on a PPI alongside palbociclib, which are consistent with the concerns raised by Lee et al.^2^

In 2020, palbociclib was reformulated (at least in some parts of the world) to change from a capsule to a tablet formulation. Pharmacokinetic data suggests that bioavailability of palbociclib may be reduced by PPIs in capsule formulation but this has not been shown to be the case with the newer tablet formulation.^3^ The formulation does not seem to be described in Chen et al.’s discussion at all. A multicenter Japanese study published in your journal also conflicts with the results reported recently.^4^

The authors also note that there were differences in their population co-morbidity scores. They suggest this is attributable to the need for a diagnosis of peptic ulcer disease to be reimbursed for a PPI in their health economy. However, they do state, “*A higher comorbidity score is associated with an increased risk of mortality, which makes it difficult to determine whether the poorer outcomes are primarily due to PPI use or the patient’s underlying health status*.” It is unclear, therefore, if correlation is causation.

Most clinicians checking for drug-drug interactions will not review the primary data source and will instead use a reference text or database, which is likely to provide a minimalist summary based on literature reviews, which, like this article, may omit to describe the formulation. Indeed, many newer oncologists and pharmacists will also be unaware of the formulation change of palbociclib, and this article makes no reference to which formulation was in use at the time of the study.

While it may be reasonable to consider the use of H2 antagonists in preference to a proton pump inhibitor out of an abundance of caution, it seems most likely that the reformulation may have already addressed the absorption issues. We are likely to continue to use CDK4/6 inhibitors for a considerable time into the future, and it would be concerning if generations to come were to simply accept there is definitively an interaction with all forms of CDK4/6 inhibitors and PPIs, perhaps unaware there was ever a previous formulation.

## References

[1] Chen B-F, Lai J-I, Tsai Y-F, et al Real-world evaluation of CDK4/6 inhibitors in hormone receptor-positive metastatic breast cancer: prognostic effect of proton pump inhibitor use. Oncologist. 2025;30:oyaf268. 10.1093/oncolo/oyaf268 40891875 PMC12505142

[2] Lee JE, Kwon SH, Kwon S, Jung HI, Nam JH, Lee EK. Concomitant use of proton pump inhibitors and palbociclib among patients with breast cancer. JAMA Netw Open. 2023;6:e2324852. 10.1001/jamanetworkopen.2023.24852 37477917 PMC10362477

[3] Schieber T, Steele S, Collins S, et al Effect of concurrent proton pump inhibitors with palbociclib tablets for metastatic breast cancer. Clin Breast Cancer. 2023;23:658-663. 10.1016/j.clbc.2023.05.009 37296062

[4] Takahashi K, Uozumi R, Mukohara T, et al Proton pump inhibitors and Cyclin-Dependent kinase 4/6 inhibitors in patients with breast cancer. Oncologist. 2024; 29:e741-e749. 10.1093/oncolo/oyae015 38340010 PMC11144975

